# Proposal of an Adapted Physical Activity Exercise Protocol for Women with Osteoporosis-Related Vertebral Fractures: A Pilot Study to Evaluate Feasibility, Safety, and Effectiveness

**DOI:** 10.3390/ijerph16142562

**Published:** 2019-07-18

**Authors:** Sofia Marini, Erica Leoni, Alessandra Raggi, Tiziana Sanna, Nazzarena Malavolta, Buffa Angela, Pasqualino Maietta Latessa, Laura Dallolio

**Affiliations:** 1 Department of Life Quality Studies, University of Bologna, Campus of Rimini, Corso d’Augusto 237, 47921 Rimini, Italy; 2 Unit of Hygiene, Public Health and Medical Statistics, Department of Biomedical and Neuromotor Sciences, University of Bologna, via San Giacomo 12, 40126 Bologna, Italy; 3 School of Hygiene and Preventive Medicine, Department of Biomedical and Neuromotor Sciences, University of Bologna, via San Giacomo 12, 40126 Bologna, Italy; 4 Sant’Orsola-Malpighi Hospital of Bologna, Unit of Internal Medicine, Section of Rheumatology, via Pietro Albertoni, 15, 40138 Bologna, Italy

**Keywords:** osteoporosis-related vertebral fractures, adapted physical activity, health-related quality of life, fear of falling, physical performance

## Abstract

A quasi-experimental pilot study was performed to determine the feasibility and safety of an Adapted Physical Activity (APA) protocol and its effect on health-related quality of life (HRQOL), fear of falling, pain, and physical performance in women with osteoporosis-related vertebral fractures. Forty-four post-menopausal women (mean age: 67.6 ± 4.6) with osteoporotic vertebral fractures were assigned to an exercise group (APA group = 26) who attended a six-month exercise protocol that included postural and muscular reinforcement exercises, and a control group (CG = 18) who was asked to maintain their current lifestyle. At baseline and six months after baseline, HRQOL was measured as primary outcome by the Assessment of Health Related Quality of Life in Osteoporosis (ECOS-16) questionnaire. Secondary outcomes were fear of falling (Fall Efficacy Scale International, FES-I), lumbar back pain (Visual Analogue Scale-VAS), functional exercise capacity (Six Minutes Walking Test-6MWT, Borg scale), balance and gait (Tinetti Scale), and flexibility of the column (Chair Sit-and-Reach). The effects of the intervention were analyzed by comparison within groups and between groups. Effect sizes (ES) were calculated using Cohen’s d. All the outcomes significantly improved in the APA group, while they remained unchanged in the CG. After adjustment for unbalanced variables, the comparison between groups showed significant effects of the intervention for ECOS-16-score, functional exercise capacity, balance, and gait. The exercise program had big effect sizes on HRQOL (ES = 1.204), fear of falling (ES = 1.007), balance (ES = 0.871), and functional exercise capacity (ES = 1.390). Good adherence (75.8%) and no injuries were observed. Due to its feasibility, safety, and effectiveness, the proposed exercise protocol can be adopted in APA programs addressed to patients with osteoporosis-related vertebral fractures.

## 1. Introduction

Osteoporosis is a disease characterized by low bone mass and structural deterioration of bone tissue, leading to bone fragility and an increased susceptibility to fractures [1]. The loss of bone is symptomatically silent and progressive, until a bone fracture or a fragility fracture (defined as a fracture resulting from a low trauma) occurs. Thus, bone fractures are the main consequences of osteoporosis both in terms of clinical, social, and financial costs [2].

The number of individuals aged ≥50 years at high risk of osteoporotic fracture, worldwide in 2010, was estimated at 158 million and it is expected to double over the next 40 years [3]. Therefore, osteoporosis and the associated fractures constitute a major public health concern. Among osteoporosis-related fractures, the vertebral ones amount to approximately 15% of the total. The experience of vertebral fracture decreases mobility and physical performance and raises social isolation, lack of self-confidence, and depression [4,5]. These physical, emotional, and social aspects have a significant impact on the deterioration of health-related quality of life (HRQOL) that can be improved by osteoporosis and anti-fracture therapy. Key components of this care are physical and pharmacologic modalities of pain control and exercises or physiotherapy to maintain spinal movement and strength [6].

Physical activity is a part of the comprehensive management of osteoporotic patients. Regular physical activity, even taken up later in life, can help older women to prevent a decline in different components of HRQOL, and even improves the enjoyment of life [7]. Exercises aimed to improve lumbar spinal mobility and optimize postural alignment and stability may contribute to prevent falls in osteoporotic patients with or without vertebral fractures [8,9]. Disease-specific exercises, adapted to the pathological conditions, can improve muscle strength, balance, and posture, all factors that minimize risk of falling and, subsequently, reduce occurrence of fractures.

Nevertheless, a Cochrane Review argued that no definitive conclusions can be made regarding the benefits of exercise for individuals with vertebral fracture [10]. Furthermore, although most guidelines for prevention and treatment of osteoporosis recommend practicing physical activity regularly, it is unclear which exercise is optimal for these patients [11].

Our aim was to draw up and test a standardized exercise program, in terms of frequency, duration, intensity, and type of exercises, targeted for women with osteoporotic vertebral fractures. Specifically, we evaluated an exercise program in accordance with the principles of Adapted Physical Activity (APA), based on group exercise protocols, designed for individuals with chronic conditions, aimed at correcting sedentary lifestyle and preventing or mitigating frailty and disability through “individualizing instruction, matching personal strengths and interests” [12,13]. Applying a quasi-experimental study design, we carried out a pilot study with the aim of evaluating the feasibility and the safety of the proposed APA program and its positive effect on HRQOL and some other related conditions such as fear of falling, pain, and physical performance.

## 2. Materials and Methods

### 2.1. Study Design and Subjects

The pilot study design was a quasi-experimental controlled 6-month trial, with non-random assignment. The sample was recruited from the Rheumatology Section of the Internal Medicine Operational Unit at the Sant’Orsola Malpighi Hospital in Bologna, Emilia Romagna Region (Italy), during daily outpatient activity. Subjects eligible for the study were post-menopausal women living at home, ambulatory, aged 60–75 years, affected by overt osteoporosis, verified by dual energy X-ray absorptiometry, with one or more vertebral fractures verified by radiography. Table 1 shows the inclusion and exclusion criteria.

After inclusion, the participants were interviewed in order to assess the presence of risk factors for osteoporosis (age, Body Mass Index, familiarity, pharmacological treatments, early menopause, amenorrhea, anorexia nervosa, dietary deficiencies in vitamin D, smoking, alcohol, physical activity). In addition, patients were evaluated for the presence of other clinical comorbidities by the Cumulative Illness Rating Scale (≥) [14], and the level of weekly physical activity by the PASE (Physical Activity Scale for the Elderly) questionnaire, which combines information on leisure, household, and work-related activity [15].

Participants were assigned to the experimental group (APA group) or to the control group (CG). The random assignment of patients to the two groups was not possible, since many women refused to participate in the experimental group for practical reasons. The control group consisted of patients who self-excluded only for organizational reasons (difficulty in reaching the gym or in participating in activities at pre-established times, family commitments). We therefore preferred enrolment on a voluntary basis, thus giving all patients the opportunity to participate in a potentially effective and presumably welcome intervention.

The experimental group undertook a protocol of APA based on 1-h group sessions twice weekly, for 6 months. The subjects of the CG were asked to maintain their current lifestyle. At baseline and after 6 months’ follow-up, both groups were tested for the HRQOL as primary outcome. Fear of falling, lumbar back pain intensity, and physical performance were evaluated as secondary outcomes, since these conditions have a considerable effect on psychological state, anxiety, and loss of security, contributing to the deterioration of the quality of life [16]. In addition, the adherence to the program was calculated as the number of sessions performed compared to the sessions proposed, and cases of abandonment due to adverse events were noted to evaluate the safety of the exercise protocol.

The Local Ethics Committee approved the study (Independent Ethics Committee, Azienda Ospedaliera di Bologna, Policlinico S. Orsola-Malpighi, ref. 143/2014/U/Sper).

Informed consent was obtained from all individual participants included in the study.

### 2.2. Intervention

Table 2 summarizes the exercise protocol undertaken by the APA group and Table S1 shows the protocol in details.

In each physical activity session, the program consisted of a 15 min warm-up (aerobic, balance, and mobility exercises), followed by a 35-min sequence of strength exercises without weights, and finally 10 min of cool-down. Each session was composed of about 20 exercises, specifically selected by the trainers, according to the aim of each session, from the total 45 exercises from which the APA protocol is made up, using a simple equipment (i.e., mats, sticks, sponge balls, elastic bands). Simple and safe exercises were chosen, with incremental phases of intensity, aimed at developing mobility and balance, improve the proprioception, maintain or increase strength in major muscle groups, and optimize postural alignment. In particular, any exercise comporting spinal flexion was avoided, since it is known that this kind of exercise could favor vertebral fractures [17,18]. The program was performed in adequately equipped gyms under the direct supervision of graduates in Sciences and Techniques of Preventive and Adapted Physical Activity (Master Degree) specifically trained for the purpose. The protocol was developed over a period of 6 months and included 3 stages of progressive intensity in relation to the improvement and evolution of the abilities achieved by the participants and their feedback. Starting from the initial number of repetitions established for each exercise, the number of repetitions was increased in series of 2/3 (for example: 8 initial repetitions were progressively increased to 10–12). Once the objective was reached, the number of series could be increased up to a maximum of 5. Generally, the rest time between series was 30 s, depending on people’s needs. Exercise intensity progression was based on the repetition number combined with the rate of perceived exertion, as measured by Borg Category Ratio 10 (CR-10) scale [19]. The trainers also played a counselling role, advising on the precautions to be taken in everyday life.

### 2.3. Assessments at Baseline and 6-Months Follow-Up

The measurements were collected by designated and appropriately trained and blinded assessors.

#### 2.3.1. Health-Related Quality of Life (HRQOL)

HRQOL was evaluated by means of two questionnaires: A specific instrument for osteoporosis, named ECOS-16 (Assessment of health-related quality of life in osteoporosis), and a generic instrument named EuroQoL (EQ-5D-3L).

The ECOS-16 is a disease-specific and validated questionnaire to be used by patients with vertebral fractures attributed to osteoporosis [20,21]. The items of the ECOS-16 are divided into four dimensions: Pain, physical function, fear of illness, and psychosocial functionality. It allows calculating a total score (from 16 to 80), a partial score for each of the four dimensions, and two partial total scores: The Physical Component Summary score (PCS: Mean of pain and physical function scores) and the Mental Component Summary score (MCS: Mean of psychosocial and fear of illness scores). Lower scores correspond to a better quality of life [22].

The 3-level version of EuroQoL (EQ-5D-3L) is a standardized questionnaire for the measurement of HRQOL and was introduced in 1990 by the EuroQoL Group [23]. It essentially consists of 2 parts: The EQ-5D descriptive system and the EQ visual analogue scale (EQ VAS). The EQ-5D-3L descriptive system includes the following five dimensions: Mobility, self-care, usual activities, pain/discomfort, and anxiety/depression. Each dimension has 3 levels: No problems, some problems, and extreme problems. The patient is asked to indicate his/her health state by ticking the box next to the most appropriate statement in each of the five dimensions. The EQ VAS records the patient’s self-rated health on a vertical visual analogue scale where the endpoints are labelled “best imaginable health state” and “worst imaginable health state”.

#### 2.3.2. Fear of Falling

*Fall Efficacy Scale-International (FES-I) questionnaire*. The subjects are called to express their degree of concern about the possibility of falling during the execution of 16 activities of daily life. The FES-I uses a four-level Likert scale, each of which corresponds to a score ranging from 1 (not at all worried) to 4 (very worried). The individual scores are added together to calculate a total score from 16 to 64 [24,25,26].

#### 2.3.3. Lumbar Back Pain

*Visual Analogical Scale (VAS)*. The subjects are asked to express the intensity of the perceived lumbar pain in a one-dimensional scale, consisting of a straight line of 10 cm in length, whose ends correspond to two opposite conditions. One extreme indicates the absence of pain and corresponds to 0, the other extreme indicates the worst pain imaginable and corresponds to 10 [27,28].

#### 2.3.4. Physical Performance

*Tinetti Performance-Oriented Mobility Assessment tool (POMA)*—better known as Tinetti’s Scale—to assess the motor performance aimed at balance and gait. It was developed by Tinetti in 1986 to identify subjects at high risk of falls and consists of two parts: Balance assessment (9 items) and gait evaluation (7 items) for a total of 16 items, corresponding to 16 movements that the subject is called to perform. The supervisor assigns to each item a score ranging from 0 to 2 on the basis of the ability to perform the required actions: 0 = maximum incapacity, 2 = maximum capacity. The scores for the two sections, balance (maximum 16) and gait (maximum 12), are first counted separately and then added together to get an overall score (maximum 28) [29].

*Six Minute Walking Test (6-MWT)* to assess the functional exercise capacity correlated to physical fitness [30,31]. This test measures the distance (in meters) that a subject can quickly walk on a flat, hard surface in a period of 6 min. It is very easy to administer and allows measuring patients’ residual functional capacity in a number of pathological conditions, including osteoporosis [32,33]. The 6-MWT was associated with the Borg CR-10 Scale of Perceived Exertion, which allows individuals to subjectively rate their level of exertion during exercise. After the 6-MWT, the subjects were invited to rate their perceived exertion [19] with a number from 0 (extremely easy) to 10 (extremely heavy).

*Chair Sit-and-Reach* to assess the lower body flexibility. This is a safe and socially acceptable test, alternative to traditional floor sit-and-reach test in older adults [34]. The subject sits on the edge of the chair. One foot must remain flat on the floor, the other leg is extended forward with the knee straight, heel on the floor, and ankle bent at 90°. With one hand on top of the other and tips of the middle fingers flush, the subject is invited to slowly reach forward toward the toes by bending at the hip, keeping the back straight, head up, and the knee straight. The position must be maintained for 2 s. The distance is measured between the tips of the fingertips and the toe. The score is recorded to the nearest 1 cm as the distance reached, either a negative or positive score.

### 2.4. Statistical Analysis

The sample size was estimated by power analysis using the ECOS-16 questionnaire for the evaluation of HRQOL in post-menopausal women with osteoporosis as a primary outcome measure of the study. From published evidence, the ECOS-16 has a standard deviation of 0.8 at final follow-up assessment and a minimal clinically important difference of 0.69, which leads to an estimate of the size of the effect as 0.863 [20]. Considering an alpha error of 0.05 and a power of at least 0.8, the minimum size of the sample is estimated in 18 patients per group, with a total of 36 patients. Power analysis was carried out with G*Power 3.1.9.2 (http://www.gpower.hhu.de).

Patients in the APA group were compared with those in the CG on socio-demographic data and outcome measures using the *t* test, Mann-Whitney test, or *χ*^2^ test, as appropriate. Changes in outcomes measures were examined separately in each study group using Mann-Whitney test. Because the study groups are expected to differ in a non-randomized study design, we used linear multiple regression to compare changes in scores at 6 months between the APA group and CG after adjusting for age, baseline score of the analyzed variable, and all significantly different variables between the 2 groups at baseline. Effect sizes (ES) were calculated using Cohen’s d [35]. All tests were two-sided with a *p* value of less than 0.05 considered as statistically significant. All the analyses were carried out using IBM SPSS Statistics version 20.0 (IBM, Armonk, NY, USA).

## 3. Results

**Figure 1:**
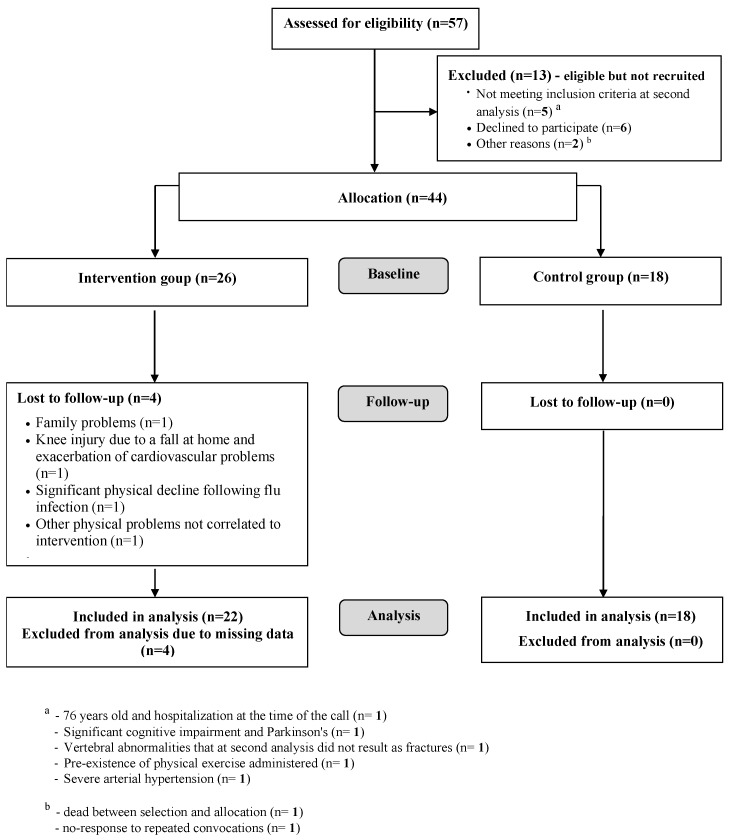
The Consort-Flowchart of participants through each stage of the trial.

A total of 57 patients were assessed for eligibility, 13 of whom were subsequently excluded from the study (Figure 1). At baseline, the study sample had 44 participants: 26 assigned to the APA group and 18 to the CG. After assignment to the intervention, four patients were lost to follow-up due to conditions arising after baseline measurements and not depending on the intervention (Figure 1). All the remaining 40 women completed the study and participated in more than 50% of sessions, 22 of the APA group and 18 of the CG. The adherence, calculated as number of sessions performed compared to the sessions proposed, was 75.8% (minimum: 56.4%; maximum: 97.8%).

Table 3 shows participants’ characteristics at baseline. The two study groups were similar in all characteristics except for the average physical activity, as measured by Physical Activity Scale for Elderly (PASE) score: CG had a significantly higher level of physical activity, in particular spent in leisure time and household activity (PASE score: Respectively, 141.8 vs. 102.3 and 58.2 vs. 25.3). Overall, at baseline, the APA group presented more risk and prognosis factors for osteoporosis than the CG, but without significant differences. Over 90% of participants had at least one co-morbidity and all 44 patients were on drug therapy for osteoporosis and did not change the pharmacological treatment throughout the intervention period.

Table 4 shows the mean scores of all primary and secondary outcomes at the beginning of the study and after six months of follow-up, and their respective mean changes from baseline. At baseline, the APA group was very disadvantaged compared with CG for most of the investigated outcomes. This finding was consistent with the difference in physical activity (PASE-score) observed between the two groups. However, while continuing to perform their general motor activities, the CG patients showed a slight worsening at follow-up and, in any case, did not improve. On the contrary, the APA group reached and exceeded the performance of the CG in HRQOL, fear of falling, and motor performance.

More specifically, HRQOL, measured by the ECOS-16 questionnaire, significantly increased in the APA group in all summary scores, whereas it remained unchanged in the CG (comparison within groups). After adjustment for age, baseline ECOS-16 and PASE, the ECOS-16 total score, “fear of illness” score, and MCS score showed statistically significant changes also in the comparison between groups. Differently, the quality of life, estimated with the generic EuroQoL VAS questionnaire, remained unchanged within and between groups.

In general, after six months of follow-up, a significant enhancement in the APA group and no changes in the CG were also found for all secondary outcomes (comparison within groups). In particular, in the APA group, the fall-related self-efficacy (FES-I) improved significantly by almost five points (*p* < 0.01) while in the CG it worsened on average by almost 1 point. These findings agree with the results obtained for lumbar back pain (APA group −1.2 points, *p* < 0.05; CG +0.3 points, ns) and the Tinetti Scale used to measure gait and balance (APA group +2.8 points, *p* < 0.01; CG: −0.7 points, ns). After adjustment for unbalanced variables, the comparison between groups maintained significant effects for the Tinetti Scale (both balance and gait subscales).

As regards the performance in motor tests, the functional exercise capacity significantly increased in the APA group (6-MWT: on average +52.2 m, *p* < 0.001), with a significant decrease of the perceived exertion (Borg Scale −1.5 points, *p* = 0.001) after the intervention. The flexibility of the column also showed an improvement in the APA group for both the right and left side (Chair Sit-and-Reach, respectively: −0.6 and −1.2). No significant differences were observed in the motor test performance of CG between baseline and follow-up. The comparison between groups confirmed the significant effects of the intervention for all motor tests, except for the right Chair Sit-and-Reach.

Table 5 shows the effect size calculated for each of the evaluated variables. According to the statistical reference parameters proposed by Cohen to interpret the results, a “big” effect (>0.8) of the intervention was observed for six outcomes (HRQOL, fear of falling, balance, functional exercise capacity, flexibility of the column at the left side) and a “medium” effect (>0.5) for four outcomes (lumbar back pain intensity, gait, perceived exertion, flexibility of the column at the right side) [35].

## 4. Discussion

The APA intervention had a significant effect on all the components of the quality of life, as measured by the disease-specific ECOS-16 questionnaire, in women with osteoporosis and vertebral fractures. In the comparison between APA group and CG, after adjustment for the confounding variables, the differences were statistically significant for the ECOS-16 total score and MCS partial score. HRQOL improvement had an effect size of 1.204 (“big” effect according to the Cohen reference) and reached the Minimal Clinically Important Difference (MCID) that must be achieved to prove an improvement in clinical status. For the ECOS-16 score, the suggested MCID is 0.5 points, representing the least improvement in general health status: “Slightly better” [20]. In contrast, the HRQOL, as measured by the generic instrument EuroQoL, did not improve after the intervention, confirming the results obtained by Papaioannou et al., who compared a disease-specific (QOQL) and a generic (Sickness Impact Profile) tool to measure HRQOL after a six-month home-based exercise program [36]. The EuroQoL questionnaire proved unsuitable for assessing the quality of life of our enrolled women, probably because the variables investigated are not discriminatory for patients who, already at baseline, had a certain degree of autonomy and mobility.

The intervention produced significant improvements for all secondary physical outcomes: Significantly higher scores were obtained for balance, gait, functional exercise capacity, perceived exertion, and flexibility. By improving physical performance, women probably increased self-esteem and self-confidence and this could have contributed to the improvement of quality of life observed for mental dimension of ECOS-16 (MCS score). For fear of falling and lumbar back pain, the APA group significantly improved after the intervention, but differences were not significant in the comparison between groups. However, the APA group, which was very disadvantaged at baseline for both conditions, strongly reduced the gap with the CG at follow up, achieving for fear of falling a big effect (1.007). For the lumbar back pain our intervention was less effective.

Very few studies are currently being carried out to evaluate the effects of exercise programs in patients with vertebral osteoporosis fractures. The most recent literature review of the Cochrane Database identifies only seven [10]. The impact of physical exercise programs on osteoporosis appears to vary depending on the frequency, duration, and intensity [37]. In accordance with our results, Bergland et al. and Evstigneeva et al. achieved beneficial and significant effects of exercise programs on the quality of life, balance, and functional mobility of patients with osteoporosis-related vertebral fractures, although using different assessment tools and physical exercise delivery times of only three months, compared with ours [38,39]. The instruments we used for the evaluation of motor performance (6-MWT, Borg scale, Chair Sit-and-Reach, Tinetti scale) are routinely applied in other fields of medicine or sports and to a lesser extent for patients with osteoporosis [29,30,31,32,33,34]. Our findings show that these tests—easy, quick, and economical to use—are suitable to evaluate the beneficial effect of physical activity even in women with osteoporosis-related vertebral fractures.

The FES-I scale was used in other studies for the measurement of fear of falling in subjects suffering from osteoporosis with or without vertebral fractures [40,41]. Olsen et al., investigating as a primary outcome the fear of falling, achieved a significant effect of exercise on the decrease of FES-I score. In our experimental conditions, the difference of FES-I score between APA group and CG was at the limit of the statistical significance (*p* = 0.059) (Table 4). This contrasting result is probably due to the smaller sample size of our study that had as primary outcome HRQOL (sample size according to power analysis: 36 subjects), while Olsen et al., using the fear of falling as primary outcome, estimated the size of the sample at 64 subjects. Nevertheless, in our study, the effect size calculated for the FES-I (1.007) was greater than that of Olsen et al. (0.4 and 0.7, respectively, after three and 12 months from baseline), which may suggest a higher appropriateness of the exercises given or an optimal duration of our intervention to reach the maximum effect [40].

Our study had an average adherence of 75.8%, higher than that of other studies of similar duration [10,36]. This is an encouraging result which, together with the satisfaction expressed by the participants, demonstrates the feasibility of the proposed APA program. The feasibility of this intervention is also ensured by the type of exercises proposed that require simple equipment (i.e., mats, sticks, sponge balls, elastic bands) and not particularly large spaces. The only specific requirement is that of personnel trained in the provision of physical exercise. It is known that adherence to exercise appears higher among studies that include supervision [10], and the role of trainers is essential to motivate and encourage participation. Another point of strength is the absence of withdrawals due to adverse events, a result that supports the adequacy and safety of the administered exercise protocol, whose intensity was calibrated on the characteristics of the patients and monitoring of their responses. According to a “patient-centered” approach, particular attention was paid to the choice of exercises, which had the objective of instructing patients to establish a workload and number of repetitions adapted to their individual functional capacity. Through feedback, the patient was educated to self-correction, to gain confidence in her abilities, to mitigate fears and hesitations in order to obtain motor autonomy.

The main limitation of the study is due to a possible selection bias related to the quasi-experimental trials, which were non-randomized studies. In order to favor the recruitment, we left the patients free to choose to participate in the intervention or control group. This approach allows for a selection bias that has been partially mitigated by the inclusion of patients referred to the same rheumatology unit, with similar demographic and clinical variables. However, the two groups were different at baseline, having the intervention group a lower level of physical activity and minor fitness compared with the control group. For this reason, in order to make the results of the two groups as comparable as possible, we applied corrective actions through an adequate statistical analysis. In the comparison between groups, we analyzed the outcomes for group differences through a multivariate analysis model, by adjusting for age, baseline PASE score, and each unbalanced variable. Non-randomization is certainly an important limit, but, in a public health context, with a view to implementing APA, it is also important to know whether an intervention can work for the patients who choose it. The patients included in our intervention group, due to non-randomization, probably represent only a part of patients with osteoporotic vertebral fractures, but having obtained beneficial effects on these women, probably more fragile than the generalized osteoporotic patients, is a result of some interest and relevance in the perspective of generalizing the pilot study intervention to a wider population.

Currently, in Italy, there is much interest and debate concerning the role of APA as a tool for prevention of chronic diseases and their consequences [42,43]. Various regional health authorities, including Emilia Romagna, have encoded protocols of APA specifically designed to provide opportunities for people with chronic diseases such as back pain, neurological disorders, and arthrosis, but not for osteoporosis with vertebral fractures [44,45,46]. The implementation of APA programs is made available to a network of gyms, uniformly distributed throughout the territory, which, after the accreditation of the regional health authorities, can administer the APA protocols of proven efficacy to chronic patients addressed by the general practitioner or the specialist doctor [45,47].

## 5. Conclusions

The purpose of this study was primarily to propose an APA program of physical exercises specifically designed for osteoporotic women with particular fragility due to vertebral fractures. The feasibility, the safety, and the positive effect of the proposed exercise protocol on quality of life, fear of falling, balance, and functional exercise capacity show that APA programs, based on protocols similar to ours, should be extended also to patients with osteoporosis and a history of vertebral fracture. The results of this study can certainly be used to support policy makers who can favor the conditions to implement APA projects in their territory, through measures included in Health Plans of Public Health Authorities. To our knowledge, the studies that reported exercise protocols for osteoporotic patients are very few [48]. The APA protocol reported here (supplementary material) may be useful for future projects to be implemented in a wider setting.

## Figures and Tables

**Table 1:** Inclusion and exclusion criteria.

Inclusion Criteria	Exclusion Criteria
Post-menopausal women; Between the ages of 60 and 75; Osteoporosis verified by dual energy X-ray absorptiometry; With or without pharmacological therapy for osteoporosis; One or more vertebral fractures verified by radiography.	1. Moderate or severe respiratory failure; 2. Recent pulmonary embolism; 3. Endocarditis, myocarditis, or recent pericarditis; 4. Advanced intermittent claudication (study of Fontaine ≥3); 5. Myocardial infarction for at least three months, or unstable angina or stress angina; 6. Heart failure > III NYHA Class; 7. Severe arterial hypertension (systolic ≥180 mmHg or diastolic ≥110 mmHg); 8. Abdominal aortic aneurysm on ultrasound (transverse caliber >3.5 cm); 9. Anomalies of the rhythm that can represent a contraindication to the performance of moderate intensity physical activity; 10. Arthrosis or fractures with severe limb limitation; 11. Paralysis or important neuromotor disorders; 12. Body Mass Index ≤18 or ≥32 kg/m^2^; 13. Neoplastic disease or with poor prognosis; 14. Pre-existence of physical exercise administered; 15. Haemoglobin <11 g/dL; 16. Other diseases that may hinder or prevent moderate intensity physical activity.

Notes: NYHA = New York Heart Association.

**Table 2:** Components of APA protocol.

Duration	Warm Up	Workout	Cool Down
	15 min	35 min	10 min
**Aim**	Cardio-respiratory conditioning, increase body temperature and metabolism, joint mobilization, upper and lower limb coordination, proprioception and postural education	Bodyweight exercises for muscular reinforcement and neuromuscular activation, increasing muscle strength and balance, without weights.	Stretching, breathing education, and muscle relaxation maintaining body awareness, collecting individual feedback on the session, in order to reacquire autonomy and active self-management
**Type of exercise**	Multi-articular exercises able to safely solicit all the main muscle groups; focus directed to joint mobilization, balance, and postural control during walking	Resistance exercise affecting all the main muscle groups was performed using isometric and dynamic bodyweight exercises.	Predominantly exercises in an upright and supine static position, able to stretch the main muscles, holding a stretch position for up to 30 s.
**Trainer’s role**	- to specify and control the right posture, breathing, and activation of the core, for each exercise - to administer only the exercises of the APA protocol without varying them and to respect the progression of workload that is established - to ensure that the intensity of the exercise does not exceed what is indicated, adapting the rhythm to the individual capacity - to keep individual case histories in mind, trying to make persons comfortable through active listening, by announcing the program of each session and explaining the objectives of the exercises of every phase - after identifying the general level of fitness, to standardize the motor learning background, since it is essential to perform the exercises by placing emphasis on the knowledge of body and the responses gradually obtained - as the motor task becomes more and more complex, to make people aware that they are working in safety by continuously monitoring their responses		

**Table 3:** Baseline characteristics of the participants, socio-demographic data, and outcome measures (*N* = 44).

Characteristics	APA Group (*n* = 26) *N* (%) or mean ± SD	CG (*n* = 18) *N* (%) or mean ± SD	*t* Test; *p*
Age	67.6 ± 4.6	67.4 ± 4.7	0.124; 0.902
Body mass index	24.7 ± 3.6	23.9 ± 3.4	0.820; 0.417
Classification of osteoporosis			
Primary	23 (82.1%)	17 (94.4%)	1.462; 0.227
Secondary	5 (17.9%)	1 (5.6%)	
Number of vertebral fractures	2.0 ± 1.2	1.8 ± 1.3	0.549; 0.586 not significant
Number of falls	3 (10.7%)	2 (11.1%)	
Osteoporosis of parents or siblings	12 (42.9%)	8 (44.4%)	0.011; 0.916
Early menopause (<45 y)	2 (7.1%)	0 (0%)	1.344; 0.246
Dietary deficiencies in vitamin D	0 (0%)	0 (0%)	-
Amenorrhea (>6 m)	0 (0%)	1 (5.6%)	1.590; 0.207
Anorexia nervosa	1 (3.6%)	2 (11.1%)	1.023; 0.312
Glucocorticosteroids	2 (7.1%)	0 (0%)	1.344; 0.246
Smokers	5 (17.9%)	1 (5.6%)	1.462; 0.227
Alcohol ^a^	0 (0%)	0 (0%)	-
Physical activity (<30 min) ^b^	13 (46.4%)	7 (38.9%)	0.253; 0.615
CIRS ^c^	27 (96,4%)	17 (94.4%)	0.104; 0.747
Severity Index	0.2 ± 0.1	0.2 ± 0.1	−0.680; 0.500
Osteoporosis medication	28 (100%)	18 (100%)	-
PASE	102.3 ± 46.6	141.78 ± 70.7	−2.286; 0.027
Leisure time activity	25.3 ± 38.4	58.2 ± 50.1	−2.515; 0.016
Household activity	74.0 ± 33.7	80.1 ± 37.7	−0.570; 0.572
Work-related activity	3 ± 7.5	3.5 ± 8.1	−0.215; 0.831

Notes: APA = Adapted Physical Activity; CG = Control Group; SD = standard deviation; CIRS = Cumulative Illness Rating Scale (maximum value = 4, minimum value = 0); PASE = Physical Activity Scale for Elderly; ^a^ ≥1 glass of wine or beer per day; ^b^ <30 min of moderate/vigorous physical activity per day; ^c^ number of patients with CIRS values ≥ 3.

**Table 4:** Outcome measures at baseline, follow-up, and change at 6 months.

Variables	APA Group (*N* = 22)				Control Group (*N* = 18)				Between Groups ^a^ *p* Value
	Baseline	Follow-Up	Change	Within Group *p* Value	Baseline	Follow-Up	Change	Within Group *p* Value	
ECOS-16	2.49 ± 0.67	2.04 ± 0.57	−0.5 ± 0.5	0.001	1.97 ± 0.61	1.98 ± 0.59	0.0 ± 0.3	0.329	0.020
Pain score	2.68 ± 0.84	2.22 ± 0.84	−0.5 ± 0.7	0.014	2.23 ± 0.98	2.22 ± 0.80	0.0 ± 0.7	0.943	0.160
Physical Function score	1.95 ± 0.60	1.55 ± 0.49	−0.4 ± 0.5	0.003	1.59 ± 0.50	1.56 ± 0.56	0.0 ± 0.4	0.630	0.120
Psychosocial score	2.36 ± 1.01	2.07 ± 0.81	−0.4 ± 0.7	0.048	1.83 ± 0.70	1.89 ± 0.73	0.1 ± 0.4	0.617	0.200
Fear of Illness score	3.59 ± 0.91	2.86 ± 1.31	−0.7 ± 1.0	0.005	2.50 ± 0.99	2.64 ± 1.25	0.1 ± 0.8	0.297	0.020
PCS	2.31 ± 0.68	1.89 ± 0.64	−0.4 ± 0.5	0.002	1.91 ± 0.69	1.89 ± 0.64	0.0 ± 0.4	0.955	0.067
MCS	2.98 ± 0.79	2.46 ± 0.88	−0.5 ± 0.6	0.002	2.17 ± 0.70	2.26 ± 0.77	0.1 ± 0.5	0.262	0.027
EuroQoL VAS	65.00 ± 18.00	70.24 ± 18.67	6.0 ± 16.6	0.126	71.11 ± 15.01	73.06 ± 18.24	1.9 ± 12.1	0.503	0.589
FES-I	29.09 ± 8.18	24.41 ± 6.71	−4.7 ± 7.4	0.006	23.83 ± 6.60	24.72 ± 8.00	0.9 ± 2.5	0.181	0.059
Lumbar back pain VAS	4.87 ± 2.33	3.65 ± 2.75	−1.2 ± 2.6	0.029	3.73 ± 2.76	4.03 ± 2.51	0.3 ± 3.3	0.758	0.719
Tinetti Scale Total	24.77 ± 5.42	27.59 ± 0.80	2.8 ± 5.2	0.003	25.83 ± 3.13	25.11 ± 3.71	−0.7 ± 2.4	0.203	0.002
Balance	14.00 ± 2.96	15.68 ± 0.65	1.7 ± 2.8	0.005	14.67 ± 1.75	14.11 ± 1.97	−0.6 ± 1.7	0.190	0.001
Gait	10.77 ± 2.56	11.91 ± 0.29	1.1 ± 2.5	0.042	11.17 ± 1.69	11.00 ± 1.85	−0.2 ± 1.4	0.606	0.014
6-MWT	395.62 ± 66.23	447.80 ± 57.31	52.2 ± 42.1	<0.001	420.52 ± 60.65	411.99 ± 56.99	−8.5 ± 45.2	0.420	<0.001
Borg Scale	3.19 ± 1.75	1.68 ± 1.09	−1.5 ± 1.5	0.001	2.75 ± 2.15	2.33 ± 1.50	−0.3 ± 2.0	0.605	0.024
Chair Sit-and-Reach right	90.19 ± 12.32	96.36 ± 1.77	6.5 ± 8.0	0.002	94.64 ± 0.44	94.00 ± 10.10	−0.6 ± 11.0	0.660	0.106
Chair Sit-and-Reach left	89.98 ± 11.22	97.05 ± 11.05	7.3 ± 7.6	0.001	94.72 ± 10.68	93.53 ± 8.89	−1.2 ± 9.3	0.831	0.026

Notes: PCS = Physical Component Summary; MCS = Mental Component Summary; VAS = Visual Analogue Scale; ^a^ Changes in measures between baseline and follow-up are compared using linear multiple regression with correction for age, baseline scores of the analyzed variable, and PASE.

**Table 5:** Effect sizes (ES) calculated using Cohen’s d.

Parameter	Effect Size (d)
6-MWT	1.390
ECOS-16	1.204
FES-I	1.007
Chair Sit-and-Reach left	1.000
Tinetti Scale Balance	0.969
Tinetti Scale Total	0.871
Chair Sit-and-Reach right	0.739
Borg Scale	0.654
Tinetti Scale Gait	0.639
Lumbar back pain VAS	0.510
EuroQoL VAS	0.276

## Supplementary Materials

The following are available online at https://www.mdpi.com/1660-4601/16/14/2562/s1, Table S1: Exercise protocol.
