# Supplementary material for: Proposal of an Adapted Physical Activity Exercise Protocol for Women with Osteoporosis-Related Vertebral Fractures: A Pilot Study to Evaluate Feasibility, Safety, and Effectiveness
Source: Int J Environ Res Public Health. 2019 Jul 18;16(14):2562. doi: 10.3390/ijerph16142562 (PMC6679098; doi:10.3390/ijerph16142562)
Supplement: Supplementary file 1 [file ijerph-16-02562-s001.pdf]

Table S1. Exercise Protocol.

No.	Exercise	Advise	Duration	Material	Aim	Variants
**1**	Walk around the perimeter of the gym	In the first lessons insist on proper walking	3/5 min	Sticks	Warm up to improve posture and balance	- slow walking with breath control<br>- fast walk<br>- walk with lunges<br>- rolled walk<br>- tandem walk
**2**	Walk with exercises for the upper and lower limbs		5 min	Balls or sticks	Warm up to improve posture, balance and coordination	- with various upper limb circumduction<br>- with upper limbs stretched along the sides, alternating elevation<br>- with upper limbs stretched along the sides open and close hands<br>- hands on shoulders, limb distension sup. forward or lateral<br>- bend knee to the chest; arm-leg coordination exercises with and without tools
**3**	Elevate and lower the shoulder stump		10 repetitions x 2 series		To improve shoulder mobility	Executable even when seated
**4**	Protraction and retraction of shoulders		10 repetitions x 2 series		To improve shoulder mobility	Executable even when seated
**5**	Hands on shoulders, circling clockwise and counterclockwise		10 repetitions x 2 series		To improve shoulder mobility	Executable even when seated
**6**	Upper limbs in candlestick position: pushed back (with the back leaning against the wall)	Attention at the lumbar spine: avoid over-lordosis	10 repetitions x 2 series		To improve shoulder mobility	Executable even when seated
**7**	Hold the shoulder-height stick, raise it above the head, extending the upper limbs. and return	Attention at the lumbar spine: avoid over-lordosis	10 repetitions x 2 series	Stick	To improve shoulder mobility	Executable even when seated
**8**	Holding the same handle, elevate the stick over the head with extended upper limbs, flex on the back of the neck, return to extended arms and finally, starting position	Back lat machine with starting position	10 repetitions x 2 series	Stick	To improve shoulder mobility	Executable even when seated
**9**	Holding the same grip, stick at shoulder height, extend and flex the arms		10 repetitions x 2 series	Stick	To improve shoulder mobility	Executable even when seated
**10**	Head flexion forward and return	To be avoided or suspended at the onset of vertigo	10 repetitions x 1 series		To improve cervical mobility	Executable even when seated
**11**	Tilt the head to the right, then to the left	Recommend to direct the ear towards the shoulder	10 repetitions x 1 series		To improve cervical mobility	Executable even when seated
**12**	Raise the leg and perform circling of the ankle		10 repetitions in clockwise direction, 10 in anticlockwise direction		To improve ankle mobility	Executable even when seated.<br>Executable even with extended lower limb
**13**	Firmly point the stick on the ground and retract the abdomen		3 repetitions for 8 seconds	Stick	To increase strength of core	Executable even when seated
**14**	Lying down: feet on the ground and bent knees. One hand placed on the abdomen and the other on the chest: breathing control	Beware of possible COPD cases		Mat	To optimize breathing	
**15**	Lying down: keeping one leg bent on the chest with the other leg extended to the floor. Keep for 10 sec. the	Attention to posture	5 repetitions x limb	Mat	Stretching	

	position and leg change					
**16**	Lying down with feet resting on the ground and bent knees: anteversion and retroversion of the pelvis	Inhale in anteversion, exhale in retroversion	5 repetitions x 3 series	Mat	To improve lumbar spinal and pelvis mobility	
**17**	Lying down with bent knees and arms stretched out, orient the legs to the right side and then left	During the movement, do not elevate the shoulder opposite to the twist	5 repetitions x 3 series (stay for a few seconds in position)	Mat	To improve lumbar spinal mobility	It can be done with the leg going to the ground, crossed over the other
**18**	Lying down: perform slight flexion of the leg by pushing the hand against the ipsilateral and contralateral knee in order to resist		8 repetitions for 5 seconds	Mat	To increase strength of core	Executable even when seated
**19**	Lying down: flex the leg to the chest, stretch it up and keep a few seconds (alternate legs)	To be avoided in subjects with low back pain	10 repetitions x limb	Mat	To increase strength of core	
**20**	Bridge: from supine with feet on the ground and knees bent, lift the buttocks upwards (in isometry)	Be careful not to accentuate the lordosis	5 repetitions for 5 seconds	Mat	To increase strength of gluteus and core	
**21**	Lying down with bent knees: crushing the sponge ball in isometry placed under the knee of the stretched leg. Alternate the lower limb	Keep the pelvis in retroversion	3 repetitions for 10 seconds x limb	Mat and sponge ball	To increase strength of quadriceps	
**22**	Lying down: feet resting on the ground and bent knees. Bring one knee at a time to the chest alternating the lower limbs with support		5 repetitions x 2 series x limb	Mat	To increase strength of core	In progression alternating in flight
**23**	Lying down: one limb flexed with foot resting on the ground, the other limb is extended. Lifting of the limb extended upwards and backwards	Attention to maintaining the retroversion of the pelvis	5 repetitions x 2 series x limb	Mat	To increase strength of core and quadriceps	
**24**	Lying down: progressively extend the knee bringing the foot up with the use of an elastic placed under the sole of the foot		5 repetitions x limb	Mat and elastic band	Stretching	Executable even when seated
**25**	Lying down: feet resting on the ground and knees bent, push the sponge ball between the knees		10 repetitions	Mat and sponge ball	To increase strength of adductors	Executable even when seated
**26**	Lying down with bent knees place a ball under the soles of the feet and roll it extending and flexing the legs		2 series of 8 extensions and 8 flexion	Mat and sponge ball	To increase strength of lower limb muscles	Executable even when seated
**27**	Self-stretching of the cervical spine bringing the chin to the neck and bringing the top of the head upward, keeping the shoulders low (imagine holding a book on the head)	Keep the position with free breathing, pay attention to those with respiratory problems	3 repetitions for 8 seconds		Stretching	Executable even when seated
**28**	Standing upright in front of the espalier: hands on the peg at shoulder level and arms extended. Move the torso forward and backward			Espalier	To improve scapulae mobility	
**29**	Standing upright in front of the espalier: hands on the peg at shoulder level and arms extended. One foot in support and another leg stretched with a hammer foot raised from the ground: adduction and lateral abduction	Keep body and pelvis still and aligned	10 repetitions x 2 series	Espalier	To increase strength of gluteus and lower limb muscles	- flexion-extension lower limb on the sagittal plane<br>- limb circling clockwise and counterclockwise
**30**	Standing upright in front of the espalier: hands on the peg at shoulder level and arms extended Alternate marching by touching the peg of the espalier with your foot		10 repetitions x 2 series	Espalier	To increase strength of lower limb muscles	
**31**	Standing upright in front of the espalier: hands on the peg at shoulder level and arms extended. Sponge ball		10 repetitions x 2 series	Espalier and sponge ball	To increase strength of lower limb muscles	

	under the toe and heel resting on the ground: push the ball					
**32**	Standing upright in front of the espalier: hands at shoulder level and arms extended, one foot completely resting on the ground and the other with toe on the first peg and heel to the ground. Both knees are stretched.		10 repetitions x 3 series x limb	Espalier	Stretching	
**33**	Standing upright in front of the espalier: hands at shoulder level, one foot completely resting on the ground, the other on the highest possible rung with flexed knee. In this position, move as close as possible to the backrest with the torso extending the leg completely to the ground	Be careful not to accentuate the lordosis The torso moves forward while remaining well aligned	5 alternate repetitions keeping the position for 6 seconds	Espalier	Stretching	
**34**	Standing upright in front of the espalier: hands at shoulder level, with arms extended. Lift up on the toes and then on the heels maintaining the position		5 alternate repetitions keeping the position for 6 seconds	Espalier	To improve balance, proprioception and strength of lower limb muscles	
**35**	Standing upright in front of the espalier. Alternate marching by touching the peg of the espalier with your foot		10 alternate repetitions x 2 series	Espalier	To improve balance, coordination and strength of lower limb muscles	- slow execution<br>- fast execution
**36**	Arms stretched out in front of his chest with a ball in his hands. Keep your back straight and your elbows out. Press the ball in your hands to develop isometric force	Pay attention to the posture. If necessary, lean on the wall.	5 repetition x 2 series keeping the position for 5 seconds	Sponge ball	To increase strength of upper limb and pectoral muscles	Same position, but instead of squeezing it between palms, work with scapulae: slide forward one hand by rolling the ball forward, while the other hand remains fixed. Scapular musculature lengthening.<br>Executable even when seated
**37**	Back leaning against the wall, arms stretched along the sides and push a sponge ball towards the wall, with the palms of the hands.<br>Maintain isometric position	Knees slightly bent to not accentuate the lordosis	5 repetition x 2 series keeping the position for 5 seconds	Sponge ball	To increase strength of upper limb	Same position but with a ball under each of the two palms and instead of crushing them towards the wall roll them downwards lowering the shoulders, then I go up again: for stretching
**38**	Back against the wall, slightly bent knees and 90-degree elbows attached to the body. Forearms rise outwards and find themselves parallel to the ground	Perceive the movement of the scapulae	10 repetition x 2 series		To increase strength of upper limb	Executable with palms facing inward or upward.<br>With / without ball
**39**	Standing upright in front of the wall: with a ball in hand, draw small circles on the wall keeping the arm extended forward	keep the shoulders lowered	20 sec per arm (10 in clockwise, 10 in anticlockwise)	Sponge ball	To increase strength of upper limb	
**40**	Hands behind the nape, stretch the back of the neck by bending the head forward		3 series keeping the position for 10 seconds		Stretching	Executable even when seated
**41**	Flex the head sideways, helping with the hand, first right then left		3 series for side keeping the position		Stretching	Executable even when seated
**42**	Squat on the wall		5 repetitions x 2 series		To increase strength of lower limb	
**43**	Supine Decubitus, lower limb extended, flex the knee by "sliding" the heel towards the gluteus		10 repetition s x 2 series		To increase strength of lower limb and core	
**44**	With the arm extended forward, gently pull the fingers towards you, palm facing forward		3 series for limb keeping the position for 10 minutes		Stretching	Executable even when seated

**45**	From the upright position with the legs spread to the same width as the shoulders, bring the right arm in front of the body, with the left hand grasp the elbow and pull to the left. Hold the position and repeat from the opposite side	3 series for limb keeping the position for 10 minutes	Stretching	Executable even when seated
